# Perineal Assessment and Repair Longitudinal Study (PEARLS): a matched-pair cluster randomized trial

**DOI:** 10.1186/1741-7015-11-209

**Published:** 2013-09-23

**Authors:** Khaled M K Ismail, Christine Kettle, Sue E Macdonald, Sue Tohill, Peter W Thomas, Debra Bick

**Affiliations:** 1 Professor of Obstetrics and Gynaecology, School of Clinical and Experimental Medicine, College of Medical and Dental Sciences, University of Birmingham, Birmingham, West Midlands, B15 2TT, UK; 2 Professor of Women’s Health Staffordshire University, Blackheath Lane, Stafford, Staffordshire ST18 0AD, UK; 3 Lead Midwife for Education, (until Dec 2012) College of Midwives, 15 Mansfield Street, London WIG 9NH, UK; 4 Midwife, West Midlands (South) Comprehensive Local Research Network, University Hospitals Coventry and Warwickshire NHS Trust, University Hospital, Clifford Bridge Road, Coventry CV2 2DX, UK; 5 Professor of Healthcare Statistics and Epidemiology, Bournemouth University, Clinical Research Unit, School of Health and Social Care, R506A Royal London House, Christchurch Road, Bournemouth, Dorset BH1 3LT, UK; 6 Professor of Evidence Based Midwifery Practice, Kings College London, Florence Nightingale School of Nursing and Midwifery, 57 Waterloo Road, London SE1 8WA, UK

**Keywords:** Childbirth, Perineum, Trauma, Cluster RCT, Quality improvement

## Abstract

**Background:**

Perineal trauma during childbirth affects millions of women worldwide every year. The aim of the Perineal Assessment and Repair Longitudinal Study (PEARLS) was to improve maternal clinical outcomes following childbirth through an enhanced cascaded multiprofessional training program to support implementation of evidence-based perineal management.

**Methods:**

This was a pragmatic matched-pair cluster randomized controlled trial (RCT) that enrolled women (n = 3681) sustaining a second-degree perineal tear in one of 22 UK maternity units (clusters), organized in 11 matched pairs. Units in each matched pair were randomized to receive the training intervention either early (group A) or late (group B). Outcomes within each cluster were assessed prior to any training intervention (phase 1), and then after the training intervention was given to group A (phase 2) and group B (phase 3). Focusing on phase 2, the primary outcome was the percentage of women who had pain on sitting or walking at 10 to 12 days post-natal. Secondary outcomes included use of pain relief at 10 to 12 days post-natal, need for suture removal, uptake and duration of exclusive breastfeeding, and perineal wound infection. Practice-based measures included implementation of evidence into practice to promote effective clinical management of perineal trauma. Cluster-level paired *t*-tests were used to compare groups A and B.

**Results:**

There was no significant difference between the clusters in phase 2 of the study in the average percentage of women reporting perineal pain on sitting and walking at 10 to 12 days (mean difference 0.7%; 95% CI −10.1% to 11.4%; *P* = 0.89). The intervention significantly improved overall use of evidence-based practice in the clinical management of perineal trauma. Following the training intervention, group A clusters had a significant reduction in mean percentages of women reporting perineal wound infections and of women needing sutures removed.

**Conclusion:**

PEARLS is the first RCT to assess the effects of a ‘training package on implementation of evidence-based perineal trauma management. The intervention did not significantly improve the primary outcome but did significantly improve evidence-based practice and some of the relevant secondary clinical outcomes for women.

**Trial registrations:**

ISRCTN28960026

NIHR UKCRN portfolio no: 4785.

## Background

Around 85% of women who have a vaginal birth sustain perineal trauma, either spontaneously or as a consequence of an episiotomy, and three-quarters of these women will require suturing to facilitate healing of the disrupted tissue [1]. Perineal trauma-related symptoms, particularly if they persist, can have a negative effect on a woman’s physical ability to mobilize and can also hinder post-natal recovery [2-4].

Evidence of how maternal morbidity arising from perineal trauma could be reduced in the shorter term is already available. Cochrane systematic reviews have consistently shown that continuous suturing of the vagina, perineal muscles, and skin using absorbable synthetic suture materials is associated with less perineal pain and less requirement for analgesia [5-7]. Moreover, rapidly absorbable synthetic sutures are less likely to be associated with the need to remove suture materials post-natally. Therefore, there is high-level evidence recommending the use of rapidly absorbable suture material for the repair of second-degree perineal trauma and episiotomy, using the continuous suturing technique whenever feasible. This evidence is incorporated into national clinical guidelines informing routine clinical care in the UK and other countries [8,9].

In the UK, trained midwives are responsible for the care of women during normal vaginal births and they also undertake any related perineal assessments and repairs. Obstetricians tend to be involved if the perineal trauma is deemed to be complex, is more than a second-degree tear, or as part of an operative vaginal birth. As part of the preliminary work for the Perineal Assessment and Repair Longitudinal Study (PEARLS), the study team conducted a comprehensive baseline national survey of a representative sample of midwives in clinical practice. The survey highlighted inadequate implementation of evidence into practice in relation to the management of childbirth-related perineal trauma, with only 6% of midwives using the recommended evidence-based suturing technique. Moreover, participants highlighted that one of the main reasons for this gap was related to training [10].

PEARLS formed the main part of a national clinical quality improvement (QI) project. The aim of PEARLS was to improve maternal clinical outcomes following childbirth through an enhanced cascaded multiprofessional training program to support implementation of evidence-based perineal management.

## Methods

### Ethics approval

The study received ethical approval from the Thames Valley ethics committee (PEARLS REC reference - 07/MRE 12/2, ISRCTN – 28960026 (http://www.controlled-trials.com/ISRCTN28960026/pearls) and NIHR UKCRN - 4785), and was conducted in accordance with the published study protocol [11].

### Participants

It was our intention to test the effectiveness of a QI intervention in increasing the implementation of evidence-based perineal assessment and repair guidance by midwives and obstetricians involved in the provision of intra-partum and post-partum care within the hospital and in the community. To minimize contamination, we used a randomized cluster design, with the unit of randomization and analysis being the maternity unit.

The study groups comprised 11 matched maternity unit pairs, and the units (clusters) within each matched pair were randomly allocated to receive the QI intervention either early (cluster A) or late (cluster B) in the study period. To ensure the generalizability of findings it was important that the study reflected differences between the different models of maternity care in workload, staffing levels, and demographics of the women using the service. An open invitation to participate in the QI project was sent nationally, via the Royal College of Midwives, to heads of midwifery who have responsibility for the management of midwifery services in the UK. Those expressing interest were requested to provide information relating to their population demographics, birth rates, and current perineal care and training provision. In total, 24 National Health Service (NHS) units expressed an interest to participate; however, 1 unit was subsequently excluded because of a delay in providing the information required for matching, thus the 11 matched pairs required for the study were selected from the remaining 23 units.

Women booked to give birth in participating units were informed of the study during the antenatal period, and additional information was made available if requested. All women who sustained a second-degree perineal tear or episiotomy during childbirth were eligible unless they were aged under 16 years, were non-English speaking, or had pregnancy loss. In line with the ethics committee’s request and to maintain the autonomy of the women in each maternity unit in deciding whether they wished to be sent study questionnaires and to have their data used in study analyses, women were only included if they provided valid written consent to participate. Informed consent was obtained prior to their discharge home, and the woman’s general practitioner (family doctor) was informed about her participation in the study.

For women who consented, the information on their parity, type of vaginal birth, and methods and materials used for repair of their perineal trauma were entered by the recruiting clinician (either a midwife or an obstetrician) on a trial data entry sheet. The women were provided with a study pack containing a covering letter, a questionnaire to be completed at 10 to 12 days post-natally, and a pre-paid reply envelope for return of the questionnaire. Women who returned the initial questionnaire received a second questionnaire and pre-paid return envelope at 3 months post-partum.

### Intervention

The PEARLS-QI intervention was an interactive multiprofessional education package aimed at enhancing the knowledge and clinical skills of midwives and obstetricians to implement evidence-based assessment and management of second-degree perineal tears and episiotomy

The content of the PEARLS-QI intervention was as follows:

•Reading material for independent study and self-directed learning.

•Copies of available evidence-based national guidelines for perineal trauma management, post-natal care ,and pain relief.

•An interactive PEARLS DVD with audiovisual material covering anatomy, basic surgical skills, systematic assessment of perineal trauma, and the technique of cutting a mediolateral episiotomy and its repair. The DVD was developed to standardize training, aid facilitators in its delivery, and to be accessed by staff, if required, to refresh core information and maintain competency.

•An information leaflet for all women who had repaired perineal trauma providing advice about self-management of their perineal wound, their general health and well-being, and advice on who to contact if they had any concerns about healing of their perineum.

The intervention was implemented and cascaded within participating units by a locally appointed PEARLS facilitator in each cluster. Facilitators attended a ‘train-the-trainers’ two-day workshop organized by the PEARLS team. To minimize risk of contamination, the study team held two separate workshops, with study facilitators invited to attend one of these, depending on whether their unit was randomized to receive the intervention early or late (groups A and B) respectively. As this was a pragmatic study, facilitators could decide how they organized implementation of the PEARLS-QI intervention within their units, with ongoing advisory support from the trial team.

### Data collection

Data were collected at three time points. Baseline demographic and obstetric data were collected prior to implementation of the PEARLS-QI intervention (phase 1). Main trial data were collected following implementation of the intervention, and the clusters randomized to receive it early were compared with the matched clusters that did not receive it at this time (phase 2). To assess sustainability of the effects of the PEARLS-QI intervention, data were then collected following implementation of the intervention in the other cluster in the matched pair (phase 3). Each woman recruited had a study entry form completed by a clinician and as described above, was given a self-complete questionnaire on discharge to be completed at 10 to 12 days post-natal, and if they returned it, a second one to be completed at 3 months post-natal. A period of 3 months was allowed for the PEARLS-QI intervention to be cascaded in all clusters. Recruitment duration varied between the matched cluster pairs, depending on the size of the cluster.

### Primary outcome

Cochrane reviews related to suturing techniques and materials for perineal repair had shown that the use of evidence-based suture techniques and materials was associated with a significant reduction in perineal pain on walking or sitting within the previous 24 hours, as measured at 10 to 12 days post-natal using a four-item scale ranging from ‘none’ to ‘severe’ [7,12]. Therefore, this was selected as the primary outcome for the study.

### Secondary outcomes

Our secondary outcomes were selected from those commonly reported in previous perineal trauma management studies and relevant Cochrane reviews [5,12]. To ensure that a woman-centered focus on QI was maintained, Delphi surveys of independent service user groups were undertaken to identify the patient-reported outcomes considered most important by women who had recently experienced perineal trauma [13].

We assessed several clinical outcomes including perineal wound infection, need for suture removal, use of pain relief during the previous 24 hours, and breastfeeding rates at 10 to 12 days post-natal. At three months post-natal, we collected data on women’s Edinburgh Postnatal Depression Scale scores, if sexual intercourse had been resumed by 9 weeks post-natal, and the women-reported poor wound-healing and breastfeeding rates. Completed questionnaires were returned to the PEARLS central office.

### Practice outcomes

We evaluated the effect of the PEARLS-QI intervention on use of evidence-based perineal assessment and management, examining in particular whether clinicians used continuous non-locking suturing for the vaginal wall and muscle layer, subcuticular suturing for the perineal skin, and rapidly absorbable polyglactin sutures [5,7,12], and whether the woman received an information leaflet advising on post-natal care of her perineal wound [14]. This was assessed using information provided in the study entry sheets.

### Sample size

In a clustered design, the effect of clustering needs to be factored into the sample size calculation by means of the intra-cluster correlation coefficient (ICC) [15]. A preliminary sample size calculation was conducted prior to the start of data collection, but was refined using data from phase 1 prior to the commencement of phase 2. The sample size calculation for phase 2 of the trial, at which time only one group of units had received the QI intervention, assumed that at 10 to 12 days, 75% of the women in the control clusters would have any pain while walking or sitting during the previous 24 hours (primary clinical outcome), and also assumed an ICC of 0.013, a significance level of 1%, and a cluster size of 40. With 16 clusters (8 pairs) this would give the study 95% power to detect a 20% reduction in primary outcome from 75% to 55% [6]. This calculation assumed no benefit in power arising from the matched-pairs design. Assuming a response rate of 60% at 10 to 12 days gave a required recruitment number of 67 women in each cluster. The additional clusters (11 matched pairs) in PEARLS would preserve the sample size should any clusters have to be withdrawn from the study.

### Matching, randomization, and allocation concealment

Once the participating units were identified, matching of paired clusters and simple randomization was undertaken at Cardiff University by a researcher involved in designing but not running of the study or analysis of the data generated from it. The statistician responsible for matching and randomization was blinded to any identifiable information about participating units. Moreover, participating units were blinded to the identity of the unit to which they were matched. Matching criteria included type of maternity unit (obstetric or midwife-led), number of births per annum, availability of a perineal repair guideline, provision of perineal repair training, and availability of post-natal perineal care information for women. Intervention allocation was based on clusters rather than individuals.

### Data management

A data entry company manually entered all data into a specialized data entry software (Snap™, version 10) [16]. To assure quality, completed questionnaires were separated into batches prior to data entry, allowing individual accountability to be assigned for each questionnaire. Initial data entry and verification was subsequently validated by checking of at least 10% of questionnaires; any errors identified resulted in the whole batch and at least two subsequent batches being fully checked.

### Statistical methods

The main data analysis was conducted using IBM SPSS Statistics (version 19) [17]. Prior to analysis data were checked statistically for outlying values and logical inconsistencies. Where data from the entry details questionnaire were missing or needed to be checked, unit facilitators were contacted and any additions or corrections were entered onto the databases. The three sets of questionnaires (entry details, 10 to 12 day questionnaire, and 3-month questionnaire) were matched within each of the study phases.

A preliminary data analysis plan was published prior to the completion of data collection [11]. This was subsequently refined by the central project team and agreed by the project steering group. Analysis was by intention to treat (ITT). No imputation methods were used. A two-sided significance level of 5% was used.

The main analysis of the primary and secondary outcomes was conducted by means of a cluster-level analysis focusing on phase 2 data as specified in the protocol [11]. Thus, the unit of analysis for the comparison between early and late interventions was the cluster (maternity unit) rather than the individual women, reflecting the fact that the maternity unit was the unit of randomization, and that the intervention was delivered to maternity units. In this way any clustering effects (that is, women in the same maternity unit tending to be more similar to each other than to women from other units) were taken into account.

Summary statistics for outcome measures were calculated for each maternity unit, and compared between the matched intervention and control clusters using the paired *t*-test (with 10 degrees of freedom unless otherwise stated). For example, the proportion of women with pain when walking or sitting in the previous 24 hours was calculated as the summary statistic for each cluster. The mean difference in the summary statistic was then compared between the intervention and control clusters using the paired *t*-test, to enable the matched cluster design to be taken into account [18,19]. If the difference in summary statistics between clusters was highly skewed, the Wilcoxon signed ranks test was used instead of the paired *t*-test. Slight discrepancies between summary statistics calculated from cluster-level data and summary statistics calculated from individual level data may arise because of variations in cluster size. This was a protocol change whereby matched-pair random effects models using MLWin software was planned [20]. This was because of later concerns about estimating between cluster variability within each cluster pair because of a relatively small number of matched clusters. Moreover, small numbers of women experiencing some of the outcome measures resulted in lack of convergence in those statistical models. Where models could be run, the paired *t*-test method tended to be conservative, and all statistically significant results using the paired *t*-test method were also significant using the random effects model (data not shown).

Maintenance of the effect of the intervention in phase 3 was tested by comparing results from phase 3 for group A clusters (9 to 12 months following implementation of the PEARLS-QI intervention) with the results from phase 2 for group B clusters (when they had not received the PEARLS-QI intervention) using paired *t*-tests. This method, which still takes into account the randomized nature of the study, was used because the group B clusters had received the intervention by the time the phase 3 outcomes were collected.

## Results

**Figure 1:**
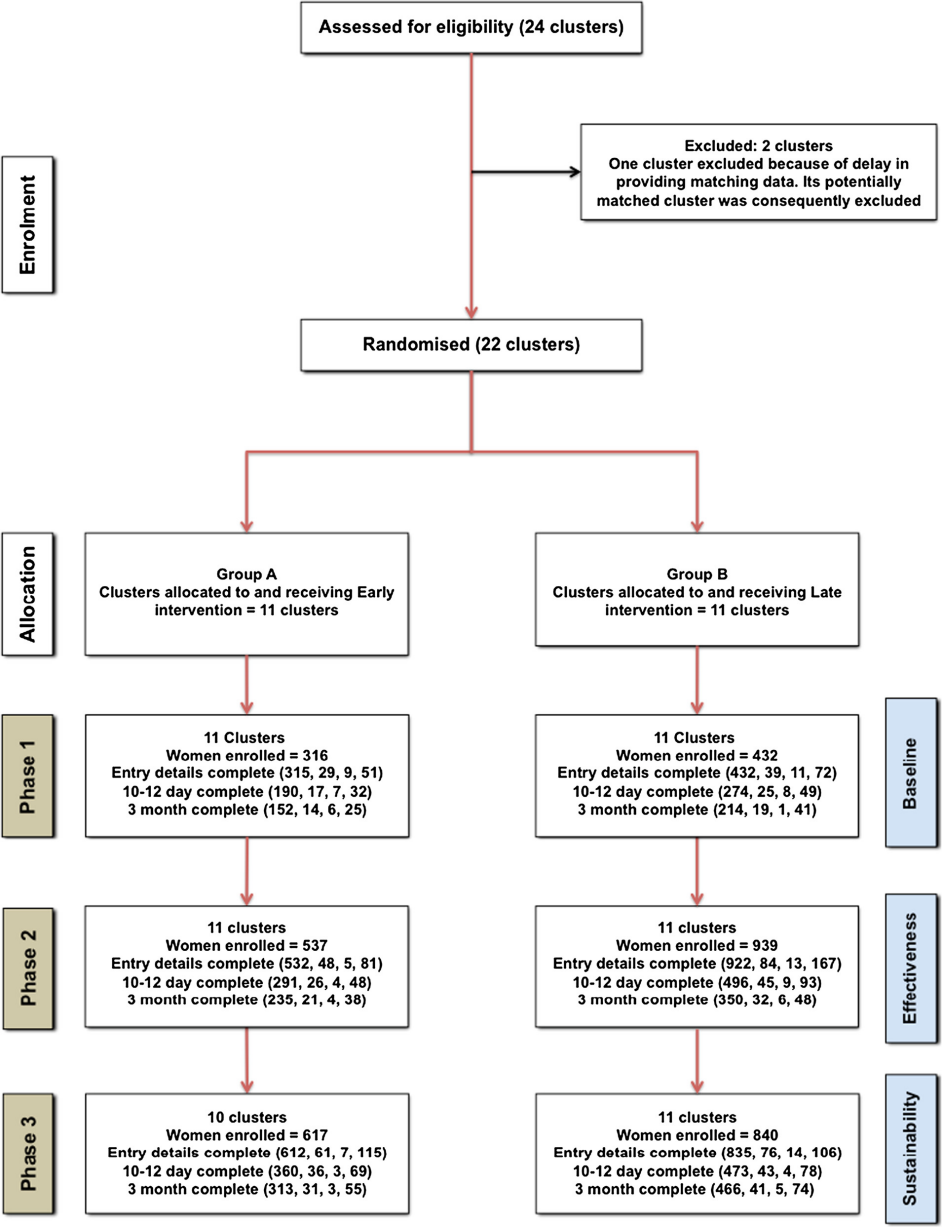
**Flow diagram of the progress of clusters and of the individuals within the clusters throughout the study.** Figures in parentheses indicate number of women, mean number of women per cluster, minimum and maximum number of women in a cluster. Phase 1: Prior to intervention implementation; Phase 2: After implementation of intervention in Group A units; Phase 3: After implementation of intervention in Group B units. In Phases 2 and 3, a period of three months was allowed for the PEARLS-QI intervention to be cascaded in all clusters. Recruitment duration varied between matched cluster pairs depending on the size of the cluster.

The results are based on data from comparison of the 11 matched paired clusters randomly allocated to receive the PEARLS-QI intervention either early or late in the study period (phase 2). The flow of women and clusters through the study is shown in Figure 1. A total of 3,681 women were recruited, with 1,470 women in group A and 2,211 in group B clusters. Based on the figures of the study-eligible women during phase 2, the overall recruitment rate was 45% (36% for group A clusters and 51% for group B clusters). Summaries of the demographic and obstetric characteristics, reported in phase 2 are presented in Table 1. In both groups combined, a total of 85 women (5.8%) did not meet the study inclusion criteria for degree of perineal trauma. One possible explanation for this is variation in classification of degree of trauma between the clinician conducting the initial examination and the clinician undertaking the repair. As this was a pragmatic RCT based on ITT, a decision was made to include the data from these women.

**Table 1:** Demographic and obstetric characteristics from phase 2 entry details in group A and group B clusters

	**Group A clusters, n =532**	**Group B clusters n = 922**
Maternal age in years, mean ± SD	28.9 ± 5.8	29.1 ± 5.7
Multiparity, % (n)	37.0% (194)	35.8% (330)
White ethnic background, % (n)	89.3% (475)	87.2% (804)
Type of delivery, % (n)		
Spontaneous vaginal	75.8% (403)	74.4% (690)
Forceps	13.9% (74)	12.9% (120)
Ventouse/suction cup	9.8% (52)	12.0% (111)
Breech	0.6% (3)	0.6% (6)
Other	0% (0)	0% (0)
Perineal trauma at delivery % (n)		
None	0.8% (4)	0.9% (8)
First-degree	1.5% (8)	2.0% (18)
Second-degree	58.9% (307)	61.6% (553)
Third-degree	3.8% (20)	3.0% (27)
Fourth-degree	0% (0)	0% (0)
Episiotomy	33.6% (175)	31.5% (283)
Extended episiotomy	1.5% (8)	1.1% (10)
Pregnancies at < 37 weeks gestation, % (n)	3.4% (18)	3.1% (29)
Birth weight, g, mean ± SD	3461 ± 485)	3460 ± 494)

The summary results of clinicians’ adherence to evidence-based practice and the women’s reported outcomes in group A and group B clusters for the three study phases are presented in Table 2 and Table 3.

**Table 2:** Descriptive statistics for the implementation of evidence-based guidelines by clinicians in group A and group B clusters in the three phases of the study

**Evidence-based standards**	**Phase 1**^**a**^		**Phase 2**^**b**^		**Phase 3**^**c**^	
	**A**	**B**	**A**	**B**	**A**	**B**
Used continuous non-locking suturing technique for vaginal wall, % (n)	56.1% (138)	65.5% (211)	78.5% (347)	67.0% (461)	72.4% (330)	77.3% (519)
Used continuous non-locking suturing technique for muscle layer, % (n)	45.2% (109)	66.1% (195)	75.1% (322)	67.3% (442)	68.1% (310)	76.8% (490)
Used subcutaneous or subcuticular suturing technique for perineal skin, % (n)	67.6% (161)	83.3% (279)	90.0% (388)	79.4% (570)	87.7% (405)	87.7% (582)
Used continuous non-locking suturing for vaginal wall and muscle layer, and used subcutaneous/subcuticular stitching for perineal skin, % (n)	35.8% (77)	56.5% (156)	72.5% (290)	57.6% (343)	64.6% (274)	73.2% (429)
Used rapidly absorbable polyglactin suturing material, % (n)	90.6% (259)	84.5% (343)	96.0% (475)	79.8% (681)	92.1% (503)	95.4% (725)
Woman received leaflet, % (n)^d^	27.7% (52)	25.6% (68)	69.1% (199)	31.4% (155)	64.5% (229)	69.3% (323)

^**a**^Prior to intervention implementation.

^**b**^After implementation of intervention in group A units, but before implementation in group B units. ^**c**^After implementation of intervention in group B units.

^**d**^Information gathered from 10 to 12 day postal questionnaire.

**Table 3:** Descriptive statistics for women-reported outcome measures in A and B clusters in phases 1, 2, and 3

	**Post-natal women-reported outcomes**	**Phase 1**^**a**^		**Phase 2**^**b**^		**Phase 3**^**c**^	
		**A**	**B**	**A**	**B**	**A**	**B**
10-12 days	Primary outcome: pain on walking or sitting in previous 24 hours, % (n)	74.5% (140)	75.6% (201)	76.7% (217)	74.1% (363)	78.5% (277)	78.2% (358)
	Total walking and sitting pain scores over previous 24 hours, mean (SD)	1.9 (1.6)	1.7 (1.4)	1.7 (1.5)	1.8 (1.5)	1.9 (1.5)	1.8 (1.5)
	Required removal of sutures, % (n)	2.1% (4)	2.2% (6)	0% (0)	3.7% (18)	1.4% (5)	2.8% (13)
	Took pain relief in previous 24 hours, % (n)	29.6% (56)	25.7% (69)	22.9% (66)	31.7% (156)	29.8% (106)	25.0% (116)
	Still breastfeeding, % (n)	65.8% (125)	66.1% (181)	63.9% (186)	67.5% (332)	68.6% (243)	69.2% (324)
	Had perineal wound infection requiring antibiotics, % (n)	6.9% (13)	5.5% (15)	2.8% (8)	6.1% (30)	5.0% (18)	3.9% (18)
3 months	Edinburgh Postnatal Depression Score ≥13, % (n)	6.7% (10)	7.6% (16)	11.2% (26)	10.1% (35)	11.8% (36)	10.6% (47)
	Resumed intercourse after 9 weeks or more, % (n)	41.9% (62)	48.6% (101)	53.3% (120)	56.1% (193)	56.0% (163)	50.7% (219)
	Poor or fairly poor perineal healing, % (n)	9.3% (14)	4.7% (10)	7.5% (17)	6.7% (23)	7.5% (23)	7.1% (31)
	Still breastfeeding, % (n)	48.3% (71)	50.0% (106)	44.8% (103)	47.6% (165)	45.2% (140)	47.4% (210)

^**a**^Prior to intervention implementation.

^**b**^After implementation of intervention in group A units, but before implementation in group B units.

^**c**^After implementation of intervention in Group B units.

There were no significant differences between group A and group B clusters with regard to pain on walking or sitting (the primary outcome measure), need for pain relief, or breastfeeding rates at 10 to 12 days post-natal. There was a significant reduction in average reported rates of wound infection (*P* = 0.03) and need for suture removal (*P* = 0.03) in group A clusters (Table 4). There was no significant difference in any of the women’s reported outcomes at 3 months post-natal.

**Table 4:** Mean differences in cluster-level summary statistics of women’s reported outcomes in phase 2

	**Post-natal outcomes**	**Mean difference**^**a **^**(95% CI)**	***P*-value**^**b**^
10-12 days	Pain walking or sitting in previous 24 hours, %	0.7% (−10.1% to 11.4%)	0.89
	Total walking and sitting pain scores over the previous 24 hours	0.10 (−0.27 to 0.46)	0.56
	Required suture removal, %^c^	2.2% (0% to 10.0%)	0.03
	Took pain relief in previous 24 hours, %	7.6% (−4.3% to 19.5%)	0.19
	Still breastfeeding, %	3.1% (−10.4% to 16.6%)	0.62
	Perineal wound infection since birth, %	4.2% (0.4% to 8.0%)	0.03
3 months	Edinburgh Postnatal Depression Score 13+, %	−1.1% (−8.1% to 6.0%)	0.75
	Resumed intercourse after 9 weeks or more, %	−3.1% (−15.9% to 9.7%)	0.60
	Poor or moderately poor perineal healing, %	0.1% (−4.9% to 5.2%)	0.95

^a^Mean difference = mean of B clusters – mean of A clusters. Positive mean differences indicate that values were on average higher in the group B (late intervention) clusters.

^b^Paired *t*-test.

^c^Highly skewed distribution, so median (95% CI) and *P*-value from Wilcoxon test are presented.

There were differences in implementation of evidence-based practice. In the trial comparison (phase 2), there was an improvement in adherence to evidence-based management of perineal trauma in the group A compared with the group B clusters. This difference was statistically significant for use of the continuous technique to repair vaginal skin (*P* = 0.007) and perineal muscles (*P* = 0.04) and in the number of perineal repairs for which the continuous suturing technique was used throughout the repair (*P* = 0.045). Women in the group A clusters were also significantly more likely to receive information about post-natal management of their perineal wounds (*P*<0.001) (Table 5).

**Table 5:** Mean differences in cluster-level summary statistics of implementation of evidence-based perineal repair in phase 2

	**Evidence-based management**	**Mean difference**^**a **^**(95% CI)**	***P*--value**^**b**^
Entry details	Use of continuous non-locking suturing technique for vaginal wall, %	−13.9% (−23.2% to −4.6%)	0.007
	Use of continuous non-locking suturing technique for muscle layer, %	−13.0% (−25.3% to −0.8%)	0.04
	Use of subcuticular suturing technique for perineal skin, %	−9.3% (−21.8% to 3.2%)	0.13
	Use of evidence-based management technique for all layers, %^c^	−16.3% (−32.1% to −0.4%)	0.045
	Use of rapidly absorbable polyglactin suture, %	−17.4% (−36.9% to 2.2%)	0.08
10 to 12 day questionnaire	Women who received post-natal leaflet, %	−39.7% (−52.9% to −26.5%)	<0.001

^a^Mean difference = mean in group B clusters – mean in group A clusters. Negative mean differences indicate higher values on average in group A (early intervention) clusters.

^b^Paired *t*-test.

^c^For this variable one cluster provided no data and so this cluster and its pair have been excluded (9 degrees of freedom).

To assess the sustainability of the PEARLS-QI intervention, the women’s reported outcomes and the use of evidence-based perineal trauma management approaches were compared between group A clusters in phase 3 (9 to 12 months after delivering the PEARLS-QI intervention) and group B clusters in phase 2 (before the delivery of the PEARLS-QI intervention). There was no significant difference between the two groups in any of the assessed outcomes except for the number of women receiving information leaflets on post-natal management of their perineal wounds (p = 0.003) (Table 6).

**Table 6:** Assessing sustainability: comparison of group A clusters in phase 3 with group B (late intervention) clusters in phase 2

	**Mean Difference (95% CI)**^**a,b**^	***P*-value**^**c**^
Sutures removed, %	1.9% (−0.8% to 8.1%)	0.18
Perineal wound infection since birth, %	1.0% (−4.0% to 6.1%)	0.66
Use of continuous non-locking suturing technique for vaginal wall, %	−3.5% (−17.2% to 10.1%)	0.57
Use of continuous non-locking suturing technique for muscle layer, %	5.1% (−9.3% to 19.5%)	0.44
Use of subcuticular suturing technique for perineal skin, %	−5.8% (−17.8% to 6.1%)	0.30
Use of evidence-based management technique for all layers, %^d^	1.7% (−16.0% to 19.4%)	0.84
Use of rapidly absorbable polyglactin suture, %	−9.4% (−32.4% to 13.7%)	0.38
Women who received post-natal leaflet, %	−34.5% (−54.2% to −14.8%)	0.003

^a^Mean difference = mean in B clusters – mean in A clusters. Negative mean differences indicate values were on average higher in the group A (early intervention) clusters.

^b^One cluster had no data at phase 3, and so this cluster and its pair were excluded (9 degrees of freedom).

^c^Paired *t*-test.

^d^For this variable one additional cluster had no data, and so this cluster and its pair were excluded (8 degrees of freedom).

## Discussion

The Institute of Medicine defines ‘quality’ as the degree to which health services for individuals and populations increase the likelihood of desired health outcomes, consistent with current professional knowledge [21].

PEARLS-QI assessed the effectiveness of implementation of an evidence-based standardized multiprofessional training package, using a matched-pair cluster RCT, on women’s health outcomes and the content of clinical practice. The gap between the availability of evidence and its implementation in relation to the management of perineal trauma following childbirth was highlighted in a national survey of midwives conducted by our team prior to designing the PEARLS-QI intervention [10]. This gap was also found in phase 1 of the study, in which prior to delivery of the QI intervention in any units, only 35.8% and 56.5% of women had perineal repairs carried out using the recommended technique in the group A and B clusters respectively. Interestingly, there was a significant improvement in the use of the continuous suturing technique for perineal repair in the group A clusters (from 35.8% to 72.5%) after delivery of the training intervention. The use of evidence-based techniques also significantly improved after delivery of the intervention in the group B clusters in phase 3 of the study. However, the improvements in the group A clusters were not sustained to the level achieved in phase 2, although this was still better than the baseline level in phase 1 (Table 2, Table 3, Table 6). Despite low rates of use of the continuous suturing technique prior to the PEARLS-QI intervention, most clinicians used the recommended more rapidly absorbed polyglactin suture material [12]. This is probably due to the fact that purchase and use of suture material tends to be decided at an organizational rather than individual clinician level. However, the potential benefits to maternal health, which may accrue from the use of appropriate suturing material, are likely to be dissipated by using less effective perineal suturing techniques. Current evidence supports the use of a continuous non-locking technique rather than interrupted suturing for perineal repair, particularly in relation to perineal pain at 10 to 12 days post-natal [5]. In spite of an improvement in the use of the recommended suturing technique, we were unable to show a reduction in women’s reported pain outcomes. This may be due to the fact that a high percentage of women in both groups (A and B) had subcutaneous sutures inserted to close the perineal skin even before delivery of the the QI intervention. Indeed, this technique of skin closure appears to be associated with a reduction in reported perineal pain [7]. Nevertheless, there was a significant reduction in rates of perineal wound infection in the group A compared with the group B clusters at the end of phase 2. Wound infection was the outcome of most importance for women in the Delphi survey we conducted during the initial project development. Genital tract sepsis was the commonest cause of direct maternal death in the UK during 2006 to 2008 [22]. Sepsis is a complex and poorly understood cause of maternal morbidity and mortality, and highlights the importance of effective care to minimize infection and the need to increase awareness of sepsis among women and clinicians.

To our knowledge, PEARLS is the first RCT to test a QI intervention specifically developed to improve use of evidence-based assessment and management of birth-related perineal trauma to reduce maternal morbidity. It is the largest study to date to evaluate the effect on women’s post-natal health of using evidence-based perineal repair methods. A major strength of the study design was the inclusion of a long-term assessment phase to measure the sustainability of the intervention. It seems that implementation of the intervention changed clinical practice, in that use of evidence-based techniques for perineal repair were better utilized several months after ‘actively’ delivering the PEARLS-QI intervention, albeit to a lesser extent compared with phase 2. We can only speculate on possible reasons for the inability to sustain the same level of improvement. It could be related to a dissipation in the effect of the training in changing attitudes, or more likely, could be related to staff service and training rotations between clinical areas causing a dilution in the number of those receiving the QI intervention and still being involved in intra-partum care. Whatever the reason, it reinforces the need for regular, ongoing updates in perineal training for those clinicians involved in intra-partum care.

There are numerous examples of delay in implementing evidence into clinical practice being associated with poor patient outcomes [23-26]. Several barriers are reported as underlying reasons for this, including lack of resources and organizational support, increased workload, and individuals’ resistance to change. In 1998, Burry and Mead suggested that to facilitate local implementation of evidence-based practice, change should be managed locally, there should be clarity about the expected benefits, and the involvement of all interested parties should be ensured [27]. PEARLS was designed as a pragmatic trial, hence in addition to testing the intervention, we wanted to ensure we used a pragmatic approach for its delivery. Therefore, the findings of our national midwifery survey helped us to understand some of the barriers and facilitators to implementation of evidence to enhance management and outcomes of perineal trauma. We believe that use of a local trained PEARLS facilitator in each cluster increased the sense of local ownership of the project and the generalizability of the study findings. Indeed, knowledge translation for healthcare professionals and consumers is more likely to be successful if the choice of translation strategy is informed by an assessment of the likely barriers and facilitators [28].

There are some limitations to our study. We did not ask facilitators to document how many of the clinicians received PEARLS training, as we considered that this would have been an additional burden and anticipated that some staff would require on-going training. As the sample size calculation was based on the primary outcome measure, there was low power for some secondary outcomes, which occurred infrequently. Over half of the women who met the study inclusion criteria were not recruited, an issue that reflects the pragmatic nature of the study, which acknowledged that service demands can compete with recruitment. This discrepancy could also be a reflection of participants’ choice because we were able to include women within a cluster only if they consented to participate. Thirdly, of the women for whom a completed entry details form was available, the percentages returning the 10 to 12 day questionnaires were 62%, 49% ,and 57% for phases 1, 2 and 3 respectively, and for the 3-month questionnaire were 49%, 40% and 53% respectively. The data analysis assumed that questionnaires are missing completely at random; bias might result if this was not the case. However, we note that response rates were comparable in the three phases and between both sets of clusters (for example, for phase 2, the 10 to 12 day questionnaire had a response rate of 54% for group A and 55% for group B, and the 3 month questionnaire had a response rate of 38% for group A and 44% for group B). Finally, the ratio of the number of women in group A clusters relative to women in group B clusters was 1.37 for phase 1, 1.75 for phase 2, and 1.36 for phase 3. The reasons for, and implications of, the ratio being greater than 1, and being greater in phase 2 compared with phases 1 and 3 are unknown, and could reflect wider individual organizational issues not addressed within the study.

There are also several strengths to our study. The risk of contamination was minimized by the use of a cluster design with matched paired maternity centers as the unit of intervention allocation. The pragmatic nature of the trial, cascading the intervention by means of local trained facilitators, and the inclusion of a range of maternity units and birth centers, increases the external validity of the study and make the findings generalizable to the UK. Additionally, publication of the trial protocol, pre-specification of the primary outcome, large sample size, and extended follow-up period were important further strengths of the study design.

In England, the Clinical Negligence Scheme for Trusts (CNST) handles all clinical negligence claims against member NHS bodies. Membership contributions are influenced by several factors, including the achievement of certain risk-management and clinical standards. In line with CNST standards, most member NHS hospitals are currently addressing clinical training provision in perineal assessment and repair to comply with CNST recommendations. Nevertheless, there is currently no standardized tested package to deliver this training or audit its effects. Similar to the implementation of the evidence-based continuous suturing technique, the improvement in women’s reported outcomes was not sustained to the same level of original improvement when assessed in phase 3. This highlights that although the PEARLS-QI intervention was effective in improving the implementation of evidence into practice, which had a positive effect on some aspects of women’s health, it is important to ensure training is actively embedded within routine clinical care to ensure that its effect is sustained. Undoubtedly, this fits in with the current model proposed by CNST, which expects that clinicians involved in intra-partum care will receive regular perineal repair and management updates. The extent to which this is currently happening is not known.

## Conclusion

The accurate assessment and appropriate repair of perineal trauma require an awareness and understanding of the supporting evidence, together with a high level of clinical skill and competency to ensure that the perineal tissues and structures are aligned correctly to promote healing and minimize morbidity.

Delivering and cascading multiprofessional training within maternity units by means of the PEARLS-QI intervention was associated with a significant improvement in adherence to evidence-based repair practice and some of the women’s reported outcomes. However, regular training updates are essential to sustain the same level of improvement. An e-learning version of the PEARLS-QI intervention is now available for online access through StratOG (the Royal College of Obstetricians and Gynaecologists e-learning resource) and the Royal College of Midwives' e-learning suite. With approximately 400,000 women sustaining perineal trauma during childbirth per annum in the UK, the clinical importance of this study cannot be underestimated, particularly if viewed in relation to its potential global benefit.

## Competing interests

KMKI and CK run perineal repair workshops both nationally and internationally, and have developed an episiotomy and second-degree tear training model with Limbs & Things, UK. Royalty fees generated from the sales of this model are managed at an organizational level, and are used to support academic activities related to women’s health. KMKI is StratOG Editor in Chief for core training modules.

## Authors’ contributions

DB and CK conceived the original idea. DB, CK, KI, and SM designed the study protocol and secured funding. ST was the PEARLS co-ordinator. PT provided statistical advice and analysis. All the authors contributed to the writing of the paper and approved the final draft.

## Pre-publication history

The pre-publication history for this paper can be accessed here:

http://www.biomedcentral.com/1741-7015/11/209/prepub
